# Combining Substrate Specificity Analysis with Support Vector Classifiers Reveals Feruloyl Esterase as a Phylogenetically Informative Protein Group

**DOI:** 10.1371/journal.pone.0012781

**Published:** 2010-09-22

**Authors:** Roberto Olivares-Hernández, Hampus Sunner, Jens C. Frisvad, Lisbeth Olsson, Jens Nielsen, Gianni Panagiotou

**Affiliations:** 1 Center for Microbial Biotechnology, Department of Systems Biology, Technical University of Denmark, Lyngby, Denmark; 2 Department of Chemical and Biological Engineering, Chalmers University of Technology, Gothenburg, Sweden; Tata Institute of Fundamental Research, India

## Abstract

**Background:**

Our understanding of how fungi evolved to develop a variety of ecological niches, is limited but of fundamental biological importance. Specifically, the evolution of enzymes affects how well species can adapt to new environmental conditions. Feruloyl esterases (FAEs) are enzymes able to hydrolyze the ester bonds linking ferulic acid to plant cell wall polysaccharides. The diversity of substrate specificities found in the FAE family shows that this family is old enough to have experienced the emergence and loss of many activities.

**Methodology/Principal Findings:**

In this study we evaluate the relative activity of FAEs against a variety of model substrates as a novel predictive tool for *Ascomycota* taxonomic classification. Our approach consists of two analytical steps; (1) an initial unsupervised analysis to cluster the FAEs substrate specificity data which were generated by cultivation of 34 *Ascomycota* strains and then an analysis of the produced enzyme cocktail against 10 substituted cinnamate and phenylalkanoate methyl esters, (2) a second, supervised analysis for training a predictor built on these substrate activities. By applying both linear and non-linear models we were able to correctly predict the taxonomic Class (∼86% correct classification), Order (∼88% correct classification) and Family (∼88% correct classification) that the 34 *Ascomycota* belong to, using the activity profiles of the FAEs.

**Conclusion/Significance:**

The good correlation with the FAEs substrate specificities that we have defined via our phylogenetic analysis not only suggests that FAEs are phylogenetically informative proteins but it is also a considerable step towards improved FAEs functional prediction.

## Introduction

Microorganisms are estimated to make up more than one-third of Earth's biomass [1]. They play essential roles in the cycling of nutrients, interact intimately with animals and plants, and directly influence Earth's climate. Fungi are one of the most ancient and diverse groups of eukaryotic organisms [2]. While their fossil remains extend back to 600 mya [3], the origin of fungi may be estimated, by phylogenetic reconstruction using a molecular clock, to be 1.5 bya or, before [4]. The recognition, delimitation and typification of fungi have been formidable problems since the discovery of these organisms. Despite the great variability in form and function- many fungi are saprobes, others are pathogens or mutualistic symbionts of plants and insects [5]- systematic studies based upon morphology and life cycle resulted, by the mid 20^th^ century, in a manageable number of major phyla.

*Ascomycota* (most yeasts and molds), *Basidiomycota* (mushrooms, smuts and rusts), *Zygomycota*, *Chytridiomycota*, and a number of taxa regarded as more ancestral forms constituted the group of organisms that were considered to be true fungi [5]. The *Ascomycota* are a large and important group of fungi, distinguished from other fungi by a saclike ascus containing haploid ascospores [5]. The *Ascomycota* encompass over 32,000 species, amounting for 40% of all described fungi [6]–[7] and its members form symbiotic, parasitic, and saprobic associations with green algae cyanobacteria and other organisms [5]. *Ascomycota* present a challenge to taxonomists because few morphological characters appear to be phylogenetically informative. Phylogenetic trees obtained from sequences of individual and multiple sets of genes do not show clustering as per class, order and family either. As a result, conflicting classification schemes have been proposed for the higher categories of *Ascomycota* [8]–[9].

Until recently, our understanding of the fungal Tree of Life has been based on two approaches, which basically differ in number of species and genes considered: (i) few genes and large number of species and (ii) large number of genes and few species. Regarding the first approach unfortunately the rationale behind the selection of these protein coding genes is not always clear, and discrepancies and incongruence between individual gene trees may result in unresolved phylogenetic trees [10]–[11]. In the second approach, a large selection of genes and/or proteins are concatenated and used for inferring phylogenetic relationships, thereby increasing the phylogenetic signal considerably [12]–[15]. However, this approach does not take into consideration the evolutionary history of each individual gene and it depends on the availability of complete genome data. Therefore, newly discovered fungi can not be easily taxonomically classified based on sequences of individual genes.

In this study we evaluate the relative activity of feruloyl esterases (FAEs, E.C. 3.1.1.73) against a variety of model substrates as a novel predictive tool of the *Ascomycota* taxonomic classification. Several fungal FAEs, responsible for cleaving the ester-link between the polysaccharides main chain of xylans and monomeric or dimeric ferulic acid, have been purified and partially characterized [16]. These enzymes exhibit different hydrolysis profiles against a wide range of model substrates, depending on: (a) changes in the type and the number of substitutions on the benzene ring, (b) changes in the distance between the ester bond and the benzene ring, and (c) change of the aliphatic region from saturated to unsaturated [17]. We examined the relationships between *Ascomycota* and FAE and to what extent the substrate specificity of FAEs is a biomarker of the known taxonomy. The described method does not depend on molecular sequences but on the whole enzymatic FAEs activity of a given fungal cell culture.

Here we explore the usefulness of the varied degree of hydrolysis towards these model substrates of FAEs to gain a more complete understanding of the relationships among fungal class, order, and family. We illustrate that the application of biostatistics and novel data-handling frameworks has a strong role in the extraction of biologically meaningful information from moderate-size data sets. In this study we demonstrate for the first time, with the use of advanced machine learning tools, that the substrate specificity of just one enzyme class, the FAEs, accurately reflects organismal relationships.

## Results and Discussion

### Evaluation of single gene phylogeny using known and putative FAEs-amino acid sequences

**Figure 1:**
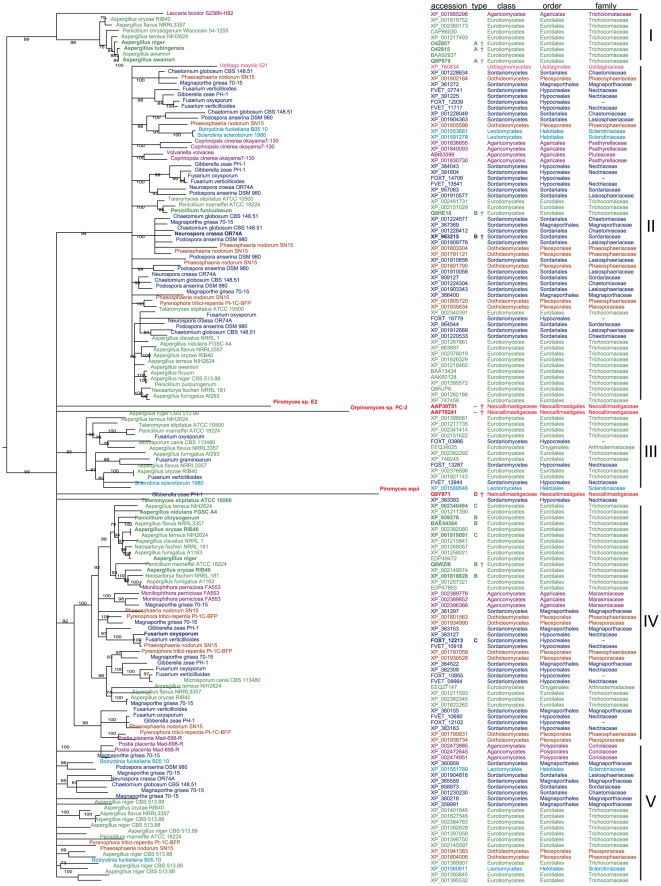
Phylogeny of putative fungal proteins selected by BLAST from NCBI and the Broad Institute using 10 known FAE amino acid sequences as the BLAST query (sequences indicated by a dagger, †). 149 unique putative proteins with a blast expect value ∼10^−40^ to any of the queries were aligned and a maximum likelihood phylogeny constructed as the consensus cladogram having at least 70% support in 100 bootstrap replicates. Bootstrap support (%) is shown at internal nodes. For each sequence, the organism, accession number and taxonomic class are shown. For biochemically characterized FAEs, the type A–D, is also indicated. Sequences have been colored according to taxonomic class.

A BLASTP search [18] was performed against all the fungal proteins in the non-redundant database of NCBI (http://blast.ncbi.nlm.nih.gov/), and the Broad Institute (http://www.broadinstitute.org/cgi-bin/annotation/fgi/blast_page.cgi/) using default parameters, with the 10 available amino acid sequences of the previously characterized fungal FAEs as input [19]. All hits above an E-value of 10^−40^ were discarded and unique and non-identical hits were retrieved. The blast analysis identified 149 unique proteins, mostly in the taxonomic classes of *Eurotiomycetes* (61) and *Sordariomycetes* (53) but several also of *Dothideomycetes* (15), *Agaricomycetes* (11), *Leotiomycetes* (5), *Neocallimastigomycetes* (3) and *Ustilaginomycetes* (1). To investigate the phylogenetic history of the putative FAEs proteins, we carried out a phylogenetic analysis of the sequences from the above representative taxonomic classes by arranging them in a Minimum Evolution Cladogram (Figure 1).

The four current classes of FAE sequences [16] have different evolutionary origin. This is evident from the cladogram of all the sequences found in the BLAST searches, which is divided into a number of distinct major clades, each with at least 85% bootstrap support. These major clades are numbered I–V from north to south.

In clade I, 7 aspergilli FAEs sequences form a well-confirmed group nested with the one sequence of the penicillium genus. This clade received bootstrap support values of 79% and 76% in two phylogenetic trees and included the three biochemically characterized members, *Aspergillus niger*, *Aspergillus awamori* and *Aspergillus tubingensis* FAEs. The latter have been classified according to Crepin *et al.*, [16] as type A (sequence similarity to lipases, induction on wheat bran, ability to hydrolyse the MFA, MSA, and MpCA, ability to release 5-5′-di-ferulic acid). Also some higher-order relationship is recovered with significant support in clade I, since all the members belong to the class of *Eurotiomycetes*. However, a putative FAE sequence from *Laccaria bicolor* belonging to the *Agaricomycetes* class shows high similarity with the above *Eurotiomycetes* sequences.

Clade II consists of an equal mix of *Sordariomycetes* (29 sequences) and sequences from each of the other main taxonomic classes, predominantly from *Eurotiomycetes* (15 sequences), intermixed with only minor clades belonging to a single taxonomic class. The clade also contains the only *Ustilaginomycetes* sequence found in this analysis. To some extent, sequences from the same taxonomic class cluster together, but there is a little intra-specific structure in the phylogeny, which means that hardly any taxonomic class-specific signatures could be attributed to the FAEs sequences. Similarly to clade I, the biochemically characterized sequences of clade II belong to a single FAE type. The FAEs from both *Penicillium funiculosum* and *Neurospora crassa* are type B (a sequence similarity to Carbohydrate Esterase Family 1, induction on sugar beet pulp, ability to hydrolyse the MFA, MpCA, and MCA, no ability to release di-ferulic acids).

The only sequence of FAE class D used as BLAST query (Q9Y871 from *Piromyces equi*) does not produce any significant hits in the blast search (data not shown) and appears in the dendrogram as a terminal node. The other sequences from the *Neocallimastigomycetes* class also appear as singular leaf nodes and are not more closely related to each other than to the rest of the sequences. These anaerobic rumen fungi are also ecologically and physiologically very different from other fungi.

The analysis of the next two clades, III and IV, demonstrate that it is not possible to determine the phylogenetic origin of a FAE based on the amino acid sequence of the protein. Clade III is composed of sequences from *Eurotiomycetes*, *Sordariomycetes* and *Leotiomycetes* in two clades supported by bootstrap values of 89% and 81%, respectively. Furthermore, as seen in Figure 1 the genes in clade III have branches longer than those of the genes in the other clades, indicating a higher rate of evolution. Clade IV has bootstrap values of 92%, 98% and 99% in the three clades and includes putative FAE from four taxonomic classes. A group of proteins from *Fusarium*, *Phaeospheria*, *Pyrenophora* and *Magnaporthe* occupies the bottom position of this clade. The additional interesting point in clade IV is the concomitant presence of sequences biochemically characterized as type B and type C. This finding indicates that information obtained by the substrate specificity and biochemical characterization of the FAE enzymes is not reflected in the primary amino acid sequence. All the biochemically characterised enzymes in the topmost part of clade IV belong to the *Eurotiomycetes*-only cluster of 15 sequences, of which four are described as FAE B sequences and two as FAE C. Together with the only characterised non-*Eurotiomycetes* sequence in the intermediate tree (FOXT_12213, FAE class C) the phylogeny shows that, for this branch, the sequence difference between the two specificities is small.

At the bottom of the phylogeny, clade V contains 24 sequences of which none have been biochemically characterised to date. This clade consists of a mix of taxonomic classes representative of the phylogeny and similar to that of clade II and IV.

Based on our analysis it could be claimed that in the most recent ancestor prior to the divergence of *Ascomycota* to different taxonomic classes there were at least four FAEs. Additionally, it is evident from the phylogenetic analysis that the amino acid sequences of FAEs is not an efficient descriptor to cluster the different *Ascomycota* strains based on their taxonomic class that they belong to.

### Functional characterization of the feruloyl esterase cocktail produced by different *Ascomycota*

**Figure 2:**
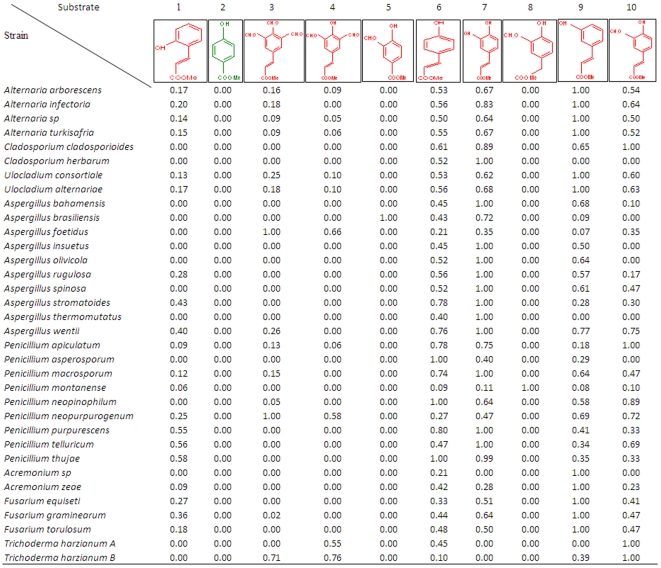
Relative feruloyl esterase activity of the 34 Ascomycota against 10 model substrates. For the substrate at which each strain shows the highest activity an arbitrary value was set to 1 and the activity against all the other substrates were scaled based on this. The red font in the structure indicates that at least one of the examined strains showed activity against this substrate, whereas, the green font indicates that none of the strains has demonstrated activity. Substrate (1): methyl-2-hydroxy cinnamate, (2): methyl-4-hydroxy benzoate, (3): methyl-3,4,5-trimethoxycinnamate, (4): methyl-4-hydroxy-3,5-dimethoxy cinnamate, (5): methyl-4-hydroxy-3-methoxy benzoate, (6): methyl-4-hydroxy cinnamate, (7): methyl-3,4-dihydroxy cinnamate, (8): methyl-4-hydroxy phenyl acetate, (9): methyl-3-hydroxy cinnamate, (10) methyl-4-hydroxy-methoxy cinnamate.

In order to test our hypothesis that the FAE specificity is a phylogenetically informative feature we selected 34 *Ascomycota* strains from our fungal collection. The strains used in this study belong to three different classes (*Dothideomycetidae*, *Eurotiomycetes*, *Sordariomycetes*), four orders (*Capnodiales*, *Pleosporales*, *Eurotiales*, *Hypocreales*), and six families (*Mycosphaerellaceae*, *Pleosporaceae*, *Trichocomaceae*, *Incertae sedis*, *Nectriaceae*, *Hypocreaceae*) while as Table 1 indicates they cover a wide range of different genera, including *Aspergillus*, *Penicillium*, *Fusarium*, *Trichoderma*, etc. All the strains were cultivated at the same FAE-inducible substrate, namely brewer's spent grain (BSG) until sufficient fungal growth was observed. Subsequently, the enzyme cocktail produced by each organism was screened for FAE enzymatic activity against a number of model substrates commonly used for this class of enzymes [17]. The characterization of each strain was based on the ability of the respective FAE cocktail to release phenolic acids from 10 substituted cinnamate and phenylalkanoate methyl esters. We use the word “cocktail” even though the specific strains evaluated in our study have not been sequenced and hence precise determination of the number of FAEs in each strain is not possible. However, a careful inspection of Figure 1 supports our assumption that more than one FAEs are present in each strain (e.g. there are 9, 7 and 2 putative FAE genes for *Aspergillus niger* CBS513.88, *Aspergillus oryzae* and *Aspergillus nidulans*, respectively, while 6 and 7 for *Fusarium oxysporum* and *Fusarium verticillioides*, respectively, and 4 for *Penicillum marneffei*), Thus the observed enzymatic activities are a combined effect of multiple FAEs and relaxed specificity of the enzymes. The structures of each substrate, which are diversified by the substitutions on the benzene ring, are shown in Figure 2. Since the optimization of the FAE production for each strain was beyond the scope of this study the values in Figure 2 indicating the enzyme activity are normalized (0–1), however, the raw data can be found in File S1. For each strain an arbitrary value of 1 was given to the substrate that the particular strain was shown the highest activity in U/mg of total protein, and the values for the rest of the substrates were scaled based on this. With the exception of methyl-4-hydroxy benzoate, indicated with green font in Figure 2, towards which no strain presented any activity against, for all the other methyl esters (red font) at least one strain was able to produce a FAE mixture that showed hydrolytic activity against the substrates. As can be observed from Figure 2, the ability to produce diverse types of FAEs, necessary to hydrolyze the esteric bond of substrates with minor structural alterations, varies for each microorganism. From the studied strains, on the on hand there were *Ascomycota* with broad FAE-substrate promiscuity, like *Alternaria arborescens*, *Ulocladium consortiale* and *Aspergillus foetidus*, which showed activity against 7 of the 10 tested substrates. On the other hand, several of the other *Aspergillus* strains presented very narrow substrate specificity with more impressive member of this group of “narrow-minded” strains the *Acremonium sp.* which could produce FAEs acting only against methyl-4-hydroxy cinnamate and methyl-3,4-dihydroxy cinnamate (Figure 2). These two substrates, in addition to methyl-3-hydroxy cinnamate and methyl-4-hydroxy-3-methoxy cinnamate could be considered as the easiest to be hydrolyzed by FAEs since almost all the strains demonstrated a general preference for them. Since there are no other reports on strain characterization based on the substrate specificity of a produced FAEs mixture, we can compare our findings only with studies on isolated FAEs. In such a study Topakas *et al.*, [17] characterized the substrate specificity of two *Fusarium*, two *Sporotrichum*, and two *Aspergillus* FAEs and in agreement with our findings concluded that, with the exception of the AnFaeA, the four aforementioned substrates contain the required substitutions on the benzene ring for correct alignment of the substrate ester bond in the FAE respective active sites so there is a preference for them.

**Table 1:** Taxonomic classification of the 34 Ascomycota used in this study.

Strain	Class	Order	Family	ID
*Cladosporium cladosporioides*	Dothideomycetes	Capnodiales	Mycosphaerellaceae	7510
*Cladosporium herbarum*	Dothideomycetes	Capnodiales	Mycosphaerellaceae	7658
*Alternaria infectoria*	Dothideomycetes	Pleosporales	Pleosporaceae	BA1209
*Ulocladium consortiale*	Dothideomycetes	Pleosporales	Pleosporaceae	9051
*Ulocladium alternariae*	Dothideomycetes	Pleosporales	Pleosporaceae	P4
*Alternaria arborescens*	Dothideomycetes	Pleosporales	Pleosporaceae	BA1342
*Alternaria turkisafria*	Dothideomycetes	Pleosporales	Pleosporaceae	BA930
*Alternaria sp.*	Dothideomycetes	Pleosporales	Pleosporaceae	8211
*Aspergillus wentii*	Eurotiomycetes	Eurotiales	Trichocomaceae	23142
*Aspergillus insuetus*	Eurotiomycetes	Eurotiales	Trichocomaceae	28265
*Aspergillus olivicola*	Eurotiomycetes	Eurotiales	Trichocomaceae	28215
*Aspergillus rugulosa*	Eurotiomycetes	Eurotiales	Trichocomaceae	22820
*Aspergillus thermomutatus*	Eurotiomycetes	Eurotiales	Trichocomaceae	28272
*Aspergillus bahamensis*	Eurotiomycetes	Eurotiales	Trichocomaceae	194726
*Aspergillus brasiliensis*	Eurotiomycetes	Eurotiales	Trichocomaceae	28178
*Aspergillus foetidus*	Eurotiomycetes	Eurotiales	Trichocomaceae	16906
*Aspergillus spinosa*	Eurotiomycetes	Eurotiales	Trichocomaceae	3022
*Aspergillus stromatoides*	Eurotiomycetes	Eurotiales	Trichocomaceae	24920
*Penicillium apiculatum*	Eurotiomycetes	Eurotiales	Trichocomaceae	18363
*Penicillium asperosporum*	Eurotiomycetes	Eurotiales	Trichocomaceae	24597
*Penicillium montanense*	Eurotiomycetes	Eurotiales	Trichocomaceae	21191
*Penicillium purpurescens*	Eurotiomycetes	Eurotiales	Trichocomaceae	22875
*Penicillium telluricum*	Eurotiomycetes	Eurotiales	Trichocomaceae	16967
*Penicillium thujae*	Eurotiomycetes	Eurotiales	Trichocomaceae	21404
*Penicillium macrosporum*	Eurotiomycetes	Eurotiales	Trichocomaceae	24758
*Penicillium neopinophilum*	Eurotiomycetes	Eurotiales	Trichocomaceae	20249
*Penicillium neopurpurogenum*	Eurotiomycetes	Eurotiales	Trichocomaceae	3933
*Acremonium sp*	Sordariomycetes	Hypocreales	Incertae sedis	9219
*Acremonium zae*	Sordariomycetes	Hypocreales	Incertae sedis	41022
*Fusarium torulosum*	Sordariomycetes	Hypocreales	Nectriaceae	8922
*Fusarium equiseti*	Sordariomycetes	Hypocreales	Nectriaceae	8752
*Fusarium graminearum*	Sordariomycetes	Hypocreales	Nectriaceae	40860
*Trichoderma harzianum A*	Sordariomycetes	Hypocreales	Hypocreaceae	9153
*Trichoderma harzianum B*	Sordariomycetes	Hypocreales	Hypocreaceae	9134

### Clustering of *Ascomycota* based on the FAEs substrate specificity

The FAE relative specificities against 10 substituted cinnamate and phenylalkanoate methyl esters were used to cluster our dataset of 34 *Ascomycota* strains. Clustering is an unsupervised data analysis technique in machine learning that attempts to group the objects based on their common traits; in this case the substrate specificity profiles. Given that there is no initial bias or predefined output, unsupervised methods are not prone to false correlations. Clustering is thus a useful technique for navigating in a dataset, evaluating its diversity and the regularities among its components and getting an overview of the information carried by the variables that describe it. The identified clusters can then form the basis for deriving a hypothesis for the development of a predictive model for new data of the same type.

**Figure 3:**
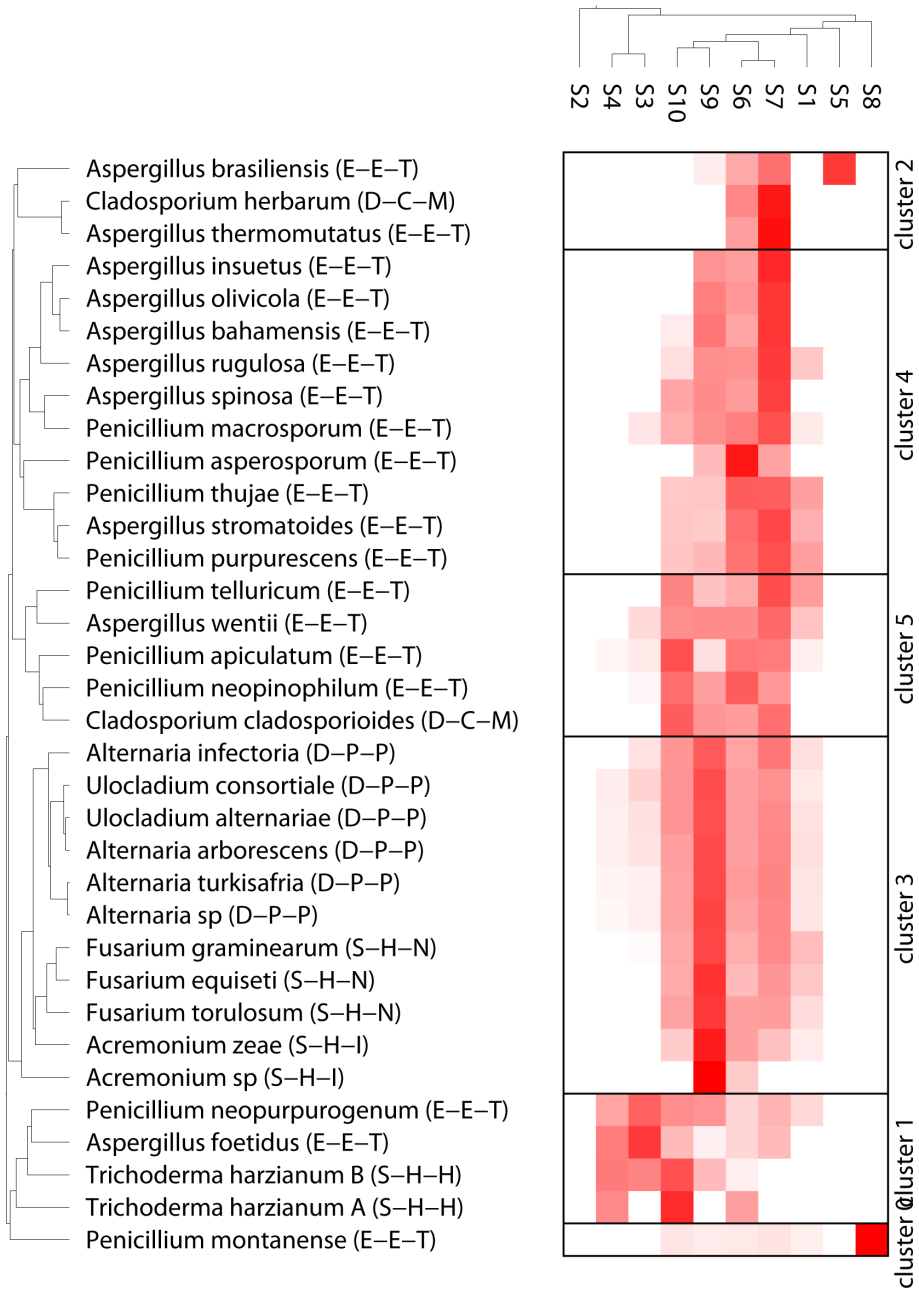
Clustering of 34 Ascomycota strains (in rows) based on the produced enzyme cocktail against 10 substituted cinnamate and phenylalkanoate methyl esters (in columns, S1 to S10 correspond to substrates as given in **Table 2**). In parenthesis next to the strain names, a three-letter code is given that corresponds to the Class-Order-Family the strain belongs to. The full names for Class, Order and Family are provided in Table 1. Different strengths of red correspond to the degree of activity on the respective substrate in relation to the activities on the rest of the substrates.

As it is shown in Figure 3, the 34 *Ascomycota* strains were distributed into 6 clusters, where each cluster has a characteristic profile of substrate specificity. **Cluster 0** contains only one strain (*Penicillium montanense*) which is characterized by specificity on substrate 8 (methyl-4-hydroxy phenyl acetate). This specie is associated to conifer trees [20], unlike the other species of *Penicillium* included in this study. **Cluster 1** contains four strains with a selective profile towards substrates 3 (methyl-3,4,5-trimethoxycinnamate), 4 (methyl-4-hydroxy-3,5-dimethoxy cinnamate) and 10 (methyl-4-hydroxy-methoxy cinnamate). Both *Trichoderma* strains are found in this cluster, whereas the other two strains belong to the same taxonomic Class, Order and Family (*Penicillium neopurpurogenum* and *Aspergillus foetidus*). **Cluster 2** is composed of three strains –two of which are the *Aspergillus brasiliensis* and *Aspergillus thermomutatus* (*Eurotiomycetes*, *Eurotiales*, *Trichocomaceae*)- that show a strong selectivity to substrate 7 (methyl-3,4-dihydroxy cinnamate). In the same cluster we also find the *Cladosporium herbarum*. **Cluster 3** is the largest of the six, with 11 members which are characterized by a wide specificity profile, with high activity on substrate 9 (methyl-3-hydroxy cinnamate) and lower activities to 6 (methyl-4-hydroxy cinnamate), 7 (methyl-3,4-dihydroxy cinnamate) and 10. This cluster can be further divided in two sub-clusters: in the north one we find the four *Alternaria* strains clustered together with the two *Ulocladium*. These six strains taxonomically belong to the same Class (*Dothideomycetes*), Order (*Pleosporales*) and Family (*Pleosporacae*). In the south sub-cluster the genus of *Fusarium* together with the genus of *Acremonium* is met. The three *Fusarium* and the two *Acremonium* strains belong to the same taxonomic Class (*Sordariomycetes*) and Order (*Hypocreales*) but different Families (*Nectriaceae* and *Incertae sedis*). Lastly, **Clusters 4** and **5** consist of the majority of *Aspergillus* and *Penicillium* strains, and the *Cladosporium cladosporioides* strain. Cluster 4 is characterized by high activity on substrate 7 and lower activities on substrates 6 and 9. Members of Cluster 5 have a wide specificity profile towards a lot of substrates, with high relative activity on at least two of them. It should be noted that Penicillia related to both *Eupenicillium* and *Talaromyces* are present in Cluster 4 and 5 despite the fact that they are phylogenetically distantly related, as they do often occupy the same plant niches [21].

From the above analysis we can conclude that there is a range of characteristic selectivity profiles of the 34 *Ascomycota* strains towards the ten tested substrates. In most cases, strains of the same taxonomic rank have very similar substrate specificity profiles. Exceptions are the *Cladosporium* strains that do not seem to share a common pattern and the *P. montanense* that forms a separate cluster, from the other *Penicillium* species. These other *Penicillium* species, and *Cladosporium* species are more prominent in deciduous forests, especially on leaf litter [21]. A dendrogram such as the one shown in Figure 3 can assist us in predicting the selectivity profile of a yet untested strain, based on its phylogenetically classification. For example, a new strain of the *Fusarium* genus is expected to have the selectivity profile of the south part of Cluster 3.

At the same time, Figure 3 gives us information about the relatedness of the substrates in respect to the FAE specificity. Substrates 6 and 7 are very similar to each other and so are substrates 9 and 10, with the level of activity against these substrates to be linked (e.g when a strain is showing lower activity against substrate 7 in comparison with another strain, it will also show lower activity against substrate 6). On the other hand, substrate 8 seems to have a very unique mode of binding, as a strain that is specific to it, does not show (significant) activity on any of the other tested substrates.

### Prediction of the class, order, and family by applying diverse classifiers

The FAE relative specificities of the 34 *Ascomycota* of Table 1 were evaluated as descriptors for developing a model to predict the taxonomic ranks (Class, Order, Family) for each strain. The observation of the natural clustering of the samples shown above was a guide towards whether it is feasible to build an accurate predictor of the taxonomy based on the given information. Four different models were used to classify the 34 organisms. The strains were grouped in different classes based on the evolutionary tree for each taxonomic category. Two categories of classifiers were applied: linear (Logistic and Linear Perceptron) and non-linear models (SMO and Multilayer perceptron). In Table 2 the performance of the linear and non-linear models is shown. The first conclusion is that the Support Vector classifier, using the relative FAE substrate activities, shows the best performance (Table 2) and the models correctly classify (>85% correct classification) the 34 strains according to the taxonomic Class, Order and Family that they belong to. In Table 2 the performance of linear and non-linear models using a MLP classifier is also compared. In the case of MLP, the linear model performs slightly better compared to the non-linear cases, with 82.4% versus 79.4% correct predictions of the taxonomic Class and 76.4% versus 67.7% correct predictions of the taxonomic Family, respectively, while no difference was observed in the prediction accuracy of the taxonomic Order (82.4%). This is not surprising since it is quite possible a problem initially thought as highly complex that can actually be solved by linear as well as non-linear techniques, like neural networks [22]–[23]. Nevertheless, these results must be taken in a conservative manner as the over-fitting using MLP models, is always possible.

**Table 2:** Performance of the different classification methods across the taxonomical categories.

	Corrected classified/misclassified		
	Class	Order	Family
**Support Vector Classifier**	29/5 (85.3%)	30/4 (88.2%)	30/4 (88.2%)
**Multilayer Perceptron (1 hidden layer)**	27/7 (79.4%)	28/6 (82.4%)	23/11 (67.7%)
**Multilayer Perceptron (no hidden layers)**	28/6 (82.4%)	28/6 (82.4%)	26/8 (76.4%)
**Logistic**	27/7 (79.4%)	25/9 (73.5%)	22/12 (64.7%)

As mentioned above, the 34 strains were classified in three different taxonomic Classes: *Dothideomycetes*, *Eurotiomycetes*, and *Sordariomycetes*. Using the SMO models, it was possible to correctly classify 29 out of the 34 strains (Table 2). The missclassfied strains belong to the *Dothideomycetes* (*Cladosporium herbarum* and *Cladosporium cladosporioides*) and *Eurotiomycetes* (*Penicillium montanense*, *Penicillium neopurpurogenum* and *Penicillium neopinophilum*) taxonomic Classes, while all the 7 *Sordariomycetes* were correctly predicted. In the case of the classification according to the taxonomic Order, the SMO model classified correctly the 30 out of the 34 strains reaching the impressive 88.2% of correct classification. The misclassified strains belong to the taxonomic Order of the *Capnodiales* (*Cladosporium herbarum* and *Cladosporium cladosporioides*) and the *Eurotiales* (*Penicillium montanense* and *Penicillium neopurpurogenum*) but a 100% correct classification was achieved for the *Pleosporales* and the *Hypocreales* strains. For the classification based on the taxonomic Order it is possible that the inability to correctly classify the *Capnodiales* strains was due to the very small number of the members of the particular Order. For the case of classifying the strains according to their taxonomic Family, the SMO model predicted again correctly the 30 out of the 34 strains. The four misclassified were again the strains *Cladosporium herbarum and Cladosporium cladosporioides* (*Mycosphaerellaceae*), followed by *Penicillium neopurpurogenum* (*Trichocomaceae*) and *Acremonium zeae* (*Incertae sedis*). In summary, it appears that the diverse substrate specificity of the FAE family is a phylogenetically informative feature. It is therefore possible to train SMO models of high accuracy, using the relative activities of FAEs, to classify strains according to their Class, Order or Family. It was also observed that *Cladosporium herbarum and Cladosporium cladosporioides* strains were constantly misclassified for all the methods of classification (see File S2). In the case of Class, the model assigned these two strains to the taxonomic Class of *Eurotiomycetes* and in the case of taxonomic Order, to the *Hypocreales*. Similarly, *Penicillium montanense* and *Penicillium neopurpurogenum* are misclassified in almost all the methods, with two exceptions (see File S2).

In this context, after having obtained initial or preliminary classification results for a given biological system, one is often left weighting the possibility of embarking on a larger and more systematic study using additional samples. While the 34 strains were carefully selected to cover most of the genus with human interest either as industrial producers of useful compounds or harmful parasites (e.g *Aspergillus*, *Penicillium*, *Fusarium*) they only belong to three namely *Dothideomycetes*, *Eurotiomycetes*, and *Sordariomycetes*, of the ten known Classes that the *Pezizomycotina* subphylum is organized. It is of great importance in order to demonstrate the broad applicability of our classification method to include members also from *Arthoniomycetes*, *Laboulbeniomycetes*, *Lecanoromycetes*, *Leotiomycetes*, *Lichinomycetes*, *Orbiliomycetes*, and *Pezizomycetes*. If we further attempt to include members grouped in a different Order for each Class and subsequently of different Family, the final number of strains will increase exponentially requiring a revision of the experimental set up. A microfermentation platform based on 96-well plates will definitively be more suitable for such an extensive study even though this cultivation approach in fungi suffers from other limitations (aeration, agitation, reproducibility, etc). However certain Orders, such as *Eurotiales* and *Dothidiales* should be well represented, as they have already given many promising candidates for enzyme production, and cover many ecological niches. Other Orders, especially the yeasts, including *Saccharomycetales* and *Schizosaccharomycetales*, are generally less chemically differentiated and produce few extracellular enzymes, and should be deemphasized. Another challenge of attempting a large scale evaluation of our hypothesis would be the high-throughput determination of the FAE activity. While in our study we used 10 model substrates to monitor the substrate specificity of FAEs there are more than 30 substrates where FAEs have demonstrated activities [17] and is general belief that there are many more to be discovered.

In addition to the biological relevance, increasing the size of the dataset is the best way to avoid overfitting of the algorithm with the rule-of-thumb to have at least 30 times as many training cases as there are weights in the network (10 times in our study); however for noise-free data, 5 times as many training cases as weights may be sufficient. Furthermore, in machine learning, building empirical scaling models called learning curves is a natural way to study classification accuracy as a function of the training set size. Learning curves estimate the empirical error rate as a function of training set size for a given classifier and dataset. These learning curves are usually well characterized by inverse power-laws:
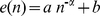
The variables are the expected error rate *e(n)* given n training samples, the learning rate *a*, the decay rate *α*, and the Bayes error *b* which is the minimum error rate achievable. The advantage of this approach is that one avoids making assumptions about the distribution generating the dataset or the distribution of the classification errors. This inverse-power-law learning behaviour appears to be universal and is observed for many classifiers and types of datasets and it could be a possible way also in our proof-of-concept study to predict the performance of the classifier when trained with additional samples (will the accuracy of the SMO improve significantly? Is the effort to collect additional samples worthwhile?).

Even though the focus of our discussion up to this point has been the prediction of the taxonomy using the different reaction specificities of FAEs, the above results can also be seen *vice versa*. In cases where the taxonomy of the strains is well defined with reliable morphological features a prediction of the functionality of the FAEs cocktail present in each strain could be made possible. During the last decade, FAEs have gained increased attention in the area of biocatalytic transformations for the synthesis of hydroxycinnamic acid esters with medicinal and nutritional applications. Feruloylation of D-arabinose by a FAE and its potential application as anti-mycobacterial agent has recently been demonstrated [24]. Furthermore, the potential of a FAE as a synthetic tool of various phenolic esters and their inhibitory effect on LDL (Low-Density-Lipoproteins) oxidation has been investigated *in vitro* in relation to prevention of atherosclerosis [25]. Since FAE synthetic activity is observed in the same structures where hydrolytic activity is feasible, our study could provide researchers and industries with an additional tool to select microbial sources of FAEs for suitable reactions and applications.

The molecular functions of enzymes are the result of a complex evolutionary interplay between environmental constraints, requirements for organismal fitness, and the functional malleability of a particular enzyme scaffold. Within these constraints, existing enzymes are recruited during evolution to perform new or modified functions, while often maintaining some aspects of the ancestral function. In addition to the study of conservation patterns in FAE sequences, we have examined the patterns of conservation and variation in enzyme function by analyzing the substrate specificity of FAEs produced by a large number of ascomycota. The clustering created by the 149 FAEs amino acid sequences retrieved from NCBI did not reflect the phylogeny of the organisms in which they occur. On the contrary our analysis on the conservation of the enzymatic activity against substrate substructures in ascomycota, focusing on the FAEs family, precisely determines aspects of taxonomy, as Class, Order and Family. As such, it provides a view of conservation and divergence different from the view afforded by more common types of studies focused on enzyme sequences and structures. While our data set of ascomycota strains and the FAEs associated substrates is relatively large, it is still limited as new reactions for this promiscuous enzyme family are discovered through the isolation and biochemical characterization of novel FAEs. In addition isolation of novel FAEs could be made based on ecological consideration based on the question: where are feruloyl esterases likely to be encountered? Furthermore, our results can be updated and improved by expanding our analysis to more fungal taxa and although it is beyond the scope of this study a valuable extension would be the correlation of the conservation patterns we see in FAEs substrates with the conservation patterns in their respective sequences. This could guide us to identify conserved motifs in the FAEs sequences that are directly connected with functional characteristics of the respective enzyme and to develop models that are able to elucidate the underlying structural characteristics that determine substrate specificities.

## Materials and Methods

### Taxonomy

All names are mentioned under their anamorph name in all tables and figures, except Fig. 1. Some of the *Penicillium* species and one *Aspergillus* species have not yet been described: *P. apiculatum*, *P. neopurpurogenum*, *P. neopinophilum*, *P. thujae*, *P. telluricum* and *Aspergillus bahamensis*. *P. apiculatum*, *P. macrosporum* ( = *Talaromyces macrosporus*), *P. neopinophilum* and *P. neopurpurogenum* are all phylogenetically closely related to *Talaromyces*, and *P. asperosporum*, *P. montanense*, *P. purpurescens*, *P. telluricum* and *P. thujae* are related to each other and the genus *Eupenicillium.Talaromyces* and *Eupenicillium* are phylogenetically very distantly related despite their common *Penicillium* genus [26]–[27]. The overall classification followed that of Kirk *et al.* [26].

### Sequence Alignment and Phylogenetic Analysis

The sequences were aligned using default parameters [29]. The phylogeny was reconstructed in PhyML version 3.0-exporté (2009-07-06) [30] using JTT as the amino acid substitution model, estimating amino acid frequency and proportion of invariable sites. One hundred bootstraps were made starting from a maximum parsimony tree. All branches with less than 70% bootstrap support were collapsed and the tree was visualized using ATV/Archaeopteryx [31]. Sequence similarity was assessed using ClustalW2, available at [http://www.ebi.ac.uk/Tools/clustalw2/].

### Strains and Cultivation Conditions

From the fungal collection at the Center for Microbial Biotechnology-DTU 34 strains (IBT numbers) were used in the present study (Table 1). The stock culture was maintained on potato-dextrose-agar (PDA) at 4°C.

A three-step cultivation procedure was employed. In the first step, each fungus was grown on PDA plates for 4 days at 30°C. In the second step, 20 ml deionised sterile water, was added to a PDA plate and aliquots (10 ml) of the spore suspension were used to inoculate Erlenmeyer flasks (250 ml) containing 20 g/L glucose and 100 ml of medium solution containing (g/l): KH_2_PO_4_ 2, (NH_4_)_2_SO_4_ 1.4, Urea 0.3, MgSO_4_ • 7H_2_O 0.3, CaCl_2_ 0.3, peptone 0.75, yeast extract 0.25, FeSO_4_ • 7H_2_O 0.005, MnSO_4_ • H_2_O 0.0015, ZnSO_4_ • 7H_2_O 0.0014, CoCl_2_ 0.002. The pH of the solution was adjusted to 5. The flasks were incubated at 30°C for 28 h in an orbital shaker (150 rpm) for mycelium production.

In the third step, an inoculum of mycelium suspension (5%, v/v) was added to the enzyme production medium in 250 ml Erlenmeyer flasks (100 ml medium) and incubated at 30°C for 6 days in an orbital shaker (200 rpm). The carbon source at this step was 20 g/L Brewer's spent grain. Cultures were carried out in duplicate.

### Chemicals and Enzyme Assays

Methyl-2-hydroxy cinnamate, methyl-4-hydroxy benzoate, methyl-3,4,5-trimethoxycinnamate, methyl-4-hydroxy-3,5-dimethoxy cinnamate, methyl-4-hydroxy-3-methoxy benzoate, methyl-4-hydroxy cinnamate, methyl-3,4-dihydroxy cinnamate, methyl-4-hydroxy phenyl acetate, methyl-3-hydroxy cinnamate, and methyl-4-hydroxy-methoxy cinnamate were purchased from Apin Chemicals Ltd. (Abingdon, UK) and Sigma-Aldrich (Denmark A/S), respectively.

The esterase activities in the supernatant of the fungal cultivations were assayed using the above substrates and the release of the acids measured by HPLC as previously described [32], except that incubations were performed at 40°C for 60 min. All assays were prepared and analyzed in duplicate, with 10% standard error for each set of results. The amount of free acid released was quantified against standard curves. One unit of activity (1U) is defined as the amount of enzyme (mg) or milliliter of culture supernatant releasing 1 µmol of free acid per minute under the defined conditions.

The total amount of protein in the supernatant was measured according to the Bradford method.

### Hierarchical Clustering

Hierarchical clustering was performed with the *vcluster* program from the Cluto clustering toolkit v.2.1.1 (http://glaros.dtc.umn.edu/gkhome/views/cluto/). The selected algorithm is based on the application of a direct clustering technique. In a direct clustering, the desired *k*-way clustering solution is computed by simultaneously finding all *k* clusters. In terms of quality, for small values of *k* as it is in our case, the direct approach leads to better clusters than those obtained by repeated bisections or the agglomerative approach.

We optimized the following clustering criterion function:
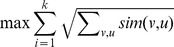
Where *k* is the total number of clusters, *S_i_* is the set of objects assigned to the *i*th cluster, *v* and *u* represent two objects and *sim(v, u)* is the similarity between two objects, which is computed using the cosine function.

The number of clusters was set to six, equal to the number of *Ascomyota* families in the dataset. This produced a good ratio between internal similarities (average similarity between the objects in each cluster) and external similarities (average similarity of the objects of each cluster and the rest of the objects).

### Classification

Four different classifiers were applied to each of the four distinct datasets where the organisms were grouped according to three taxonomic categories: Class, Order and Family. The classifiers were selected from the WEKA toolbox for the classification procedure [33]. As an exploratory strategy we applied two non-linear classifiers: Multilayer Perceptron and Sequential Minimal Optimization (SMO); and two linear classifiers: Logistic regression and Multilayer Perceptron in a linear form.

Multilayer Perceptron is a back-propagation artificial neural network. Two scenarios can be covered in the network, linear models if zero hidden layers are applied and non-linear models for 1 hidden layer or more. The optimal parameters for Multilayer Perceptron are shown in Table 3. Learning rate is the parameter that controls the step size when weights of the hidden neurons are iteratively adjusted, and the momentum is the weight applied during updating which, sometimes, gives the ability to escape from local minimum. Logistic regression is a multinomial regression model and it is used for prediction by fitting data to a logistic curve.

**Table 3:** Optimal parameters for the Multilayer Perceptron for the different taxonomic categories.

	Class	Class	Order	Order	Family	Family
**Training time**	200	200	200	200	200	200
**Learning rate**	0.5	0.3	0.3	0.3	0.5	0.3
**Momentum**	0.2	0.2	0.2	0.2	0.2	0.2
**Hidden layers/neurons**	1/6	zero	1/8	zero	1/8	zero
**Validation method**	Leave one-out cross validation	Leave one-out cross validation	Leave one-out cross validation	Leave one-out cross validation	Leave one-out cross validation	Leave one-out cross validation

SMO is a non linear method developed for training Support Vector Machines [34]. Table 4 lists the optimal parameters for this classifier. The complexity coefficient is an upper limit that restricts the influence of the support vectors on the shape of the regression function controlling the trade-off between the prediction error ε (which it is a fix value of 1E-12) and the tube's flatness. Polynomial kernel was used to train the support vector machines.

**Table 4:** Optimized parameters for the Sequential Minimal Optimization (SMO) for the different taxonomic categories.

	Class	Order	Family
Complexity parameter:	3	3	3
Kernel	Polynomial	Polynomial	Polynomial
Exponent	2	2	2
Validation method	Leave one-out cross validation	Leave one-out cross validation	Leave one-out cross validation

There is no consensus regarding which is the best cross-validation method, therefore, for every classifier two methods of validation were compared as part of the classification procedure, leave-one-out (LOO) cross-validation and 10 fold cross-validation (TF). Both are holdout methods that aim to reserve a fraction of the data for testing and use the rest for training. LOO omits one data object from the training set at each step of the training whereas TF holds 1/10 of the data. The omitted object is used to validate the performance of the model constructed from the remaining ones.

## Supporting Information

File S1 Feruloyl esterase activity in mU/mg of total protein of the 34 Ascomycota against a number of cinnamate and phenyl alkanoate methyl esters.
(0.03 MB XLS)

File S2 The table shows the strains that were misclassified (in red) and correctly classified across the different taxonomies and classifiers. These results are based on Table 5.
(0.11 MB DOC)
