# Supplementary material for: Combining Substrate Specificity Analysis with Support Vector Classifiers Reveals Feruloyl Esterase as a Phylogenetically Informative Protein Group
Source: PLoS One. 2010 Sep 22;5(9):e12781. doi: 10.1371/journal.pone.0012781 (PMC2943907; doi:10.1371/journal.pone.0012781)
Supplement: File S2 — The table shows the strains that were misclassified (in red) and correctly classified across the different taxonomies and classifiers. These results are based on Table 5. (0.11 MB DOC) [file pone.0012781.s002.doc]

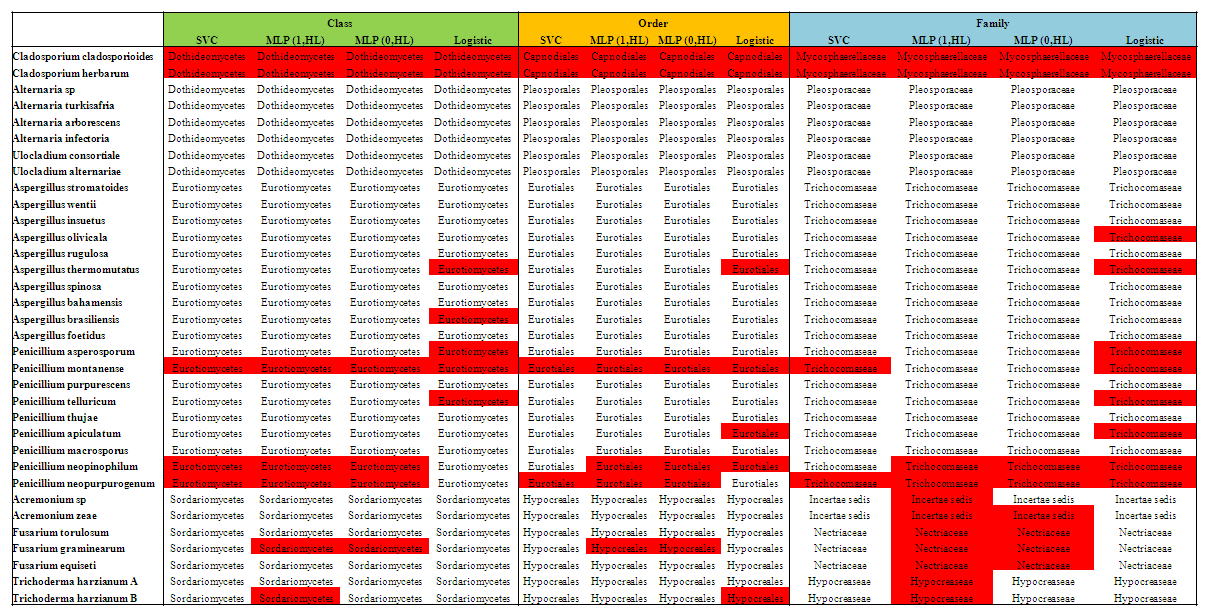


***SVC,Suport Vector Classifier; MLP, Multilayer Perceptron; (1,HL), one hidden layers; (0,HL), zero hidden layers**
